# The Case of Emperor Frederick III

**Published:** 1889-02

**Authors:** 


### The Case of Emperor Frederick III; with full reports by the German Physicians and by Sir Morell Mackenzie. New York: Edgar S. Werner. 1888.

This volume gives a full and complete report of the treatment of this much-talked-of case. Taking either way, whether we credit the tale of Dr. Mackenzie or that of the German physicians, no fame is added to old medicine by this case. It illustrates both the poverty and weakness of the school and the littleness and meanness of its practitioners. The record is well worth a careful perusal, especially by those of the homœopathic school (particularly the surgeons) who would adopt allopathic means and measures. The record of this illustrious case, as well as those of Garfield, Grant, and Conkling in this country, has revealed to the world a profession lacking knowledge and ability for all curative purposes; they can issue bulletins couched in meaningless scientific phrases; they can talk learnedly; they can use microscope and scalpel, but cure—*never*.

